# Observation of Time Reversed Light Propagation by an Exchange of Eigenstates

**DOI:** 10.1038/s41598-018-20577-w

**Published:** 2018-02-01

**Authors:** Martin Wimmer, Ulf Peschel

**Affiliations:** 1 Erlangen Graduate School in Advanced Optical Technologies (SAOT), 91058 Erlangen, Germany; 2 Institute of Solid State Theory and Optics, Abbe Center of Photonics, Friedrich-Schiller-Universität Jena, Max-Wien-Platz 1, 07743 Jena, Germany

## Abstract

As time flow dictates all evolution, its effective reversal is a topic of active research in a broad range of disciplines, including acoustics, hydrodynamics and optics. This multifarious set of environments is reflected by a great diversity of approaches to observe various echoes of wave functions. Here, we experimentally demonstrate time reversal of a pulse sequence propagating through a photonic mesh lattice realized by two coupled loops of telecommunication fibres. Our system features a symmetric band structure, which allows for almost perfect reversal of its evolution by exchanging the population between two opposing bands. The protocol applied is based on a non-adiabatic and instantaneous exchange of eigenstates resulting in highly efficient time reversal of a pulse chain.

## Introduction

Time reversal allows undoing an unwanted evolution thus restoring otherwise lost information. Therefore, manifold techniques were developed to generate time reversed signals in electromagnetic^1^, acoustic^2^, elastic^3^, hydrodynamic^4,5^ and quantum^6^ systems. By using e.g. a boundary consisting of a combination of receivers and senders, an arbitrary wave front can be synthesized, which counter propagates with respect to the initial source^2^. In optics nonlinear phenomena as three and four wave mixing allow for the creation of conjugated waves performing a backward evolution in time^7–12^. But a so-called instantaneous time mirror can also be created in a linear system by an abrupt and global change of the wave velocity, thus generating nearly perfect “echoes” of highly complex signals^13^. Time mirrors can be interpreted as a perfect imaging even through a disordered system. In waveguide arrays, this phenomenon was demonstrated by inducing the coupling to a conjugated eigenmode by a sudden phase shift of $\pi$^14,15^. More generally, dynamics can be reversed by a time dependent modulation which couples states with reciprocal evolution as e.g. forward and backward propagating waves^16–18^ or energy bands with opposing properties. In graphene, the symmetry of the band structure around Dirac points can be exploited by opening a band gap resulting in Rabi oscillations, an inversion of the carrier distribution and a final time reversal^6^. Induced transitions between bands can be adiabatic^19,20^ or non-adiabatic^11,16–18^ provided that evolution continues in a state, the propagation properties of which are inverse to the initial one.

**Figure 1:**
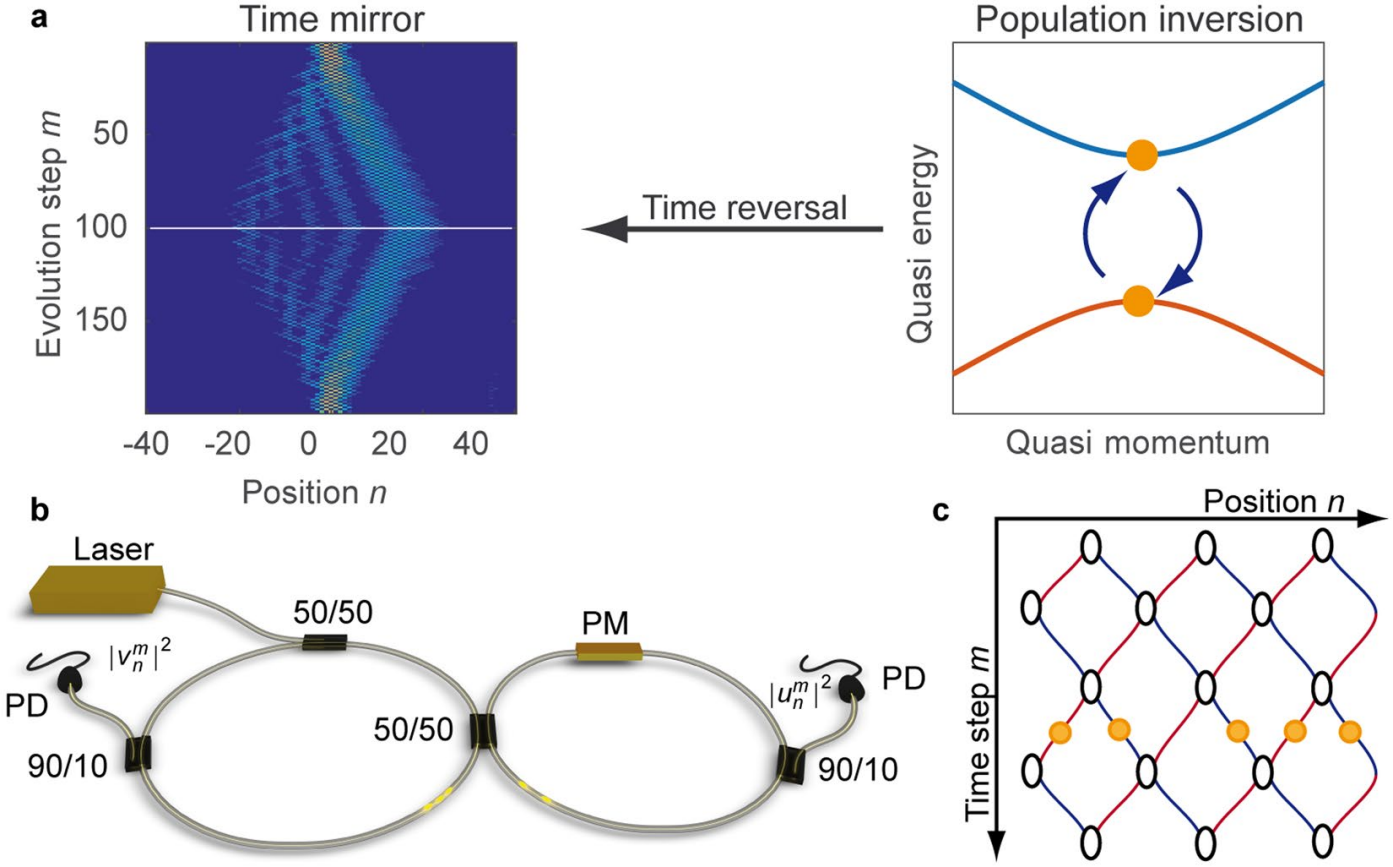
(**a**) Sketch of a time mirror. Complex wave propagation (left) is reversed by exchanging the eigenstates of a symmetric two band system (right). (**b**) The experimental set-up consists of two mutually coupled fibre loops of slightly different length, two monitor couplers with photodiodes (PD) and a phase modulator (PM) in the short loop. Pulses in the long (short) loop are labelled by ${v}_{n}^{m}$ (${u}_{n}^{m}$), where $m$ denotes the roundtrips and $n$ the difference between the number of roundtrips in the long and short loop. (**c**) The propagation through the fibre loops can be mapped on a mesh lattice spanned by $m\,\,$and $n$. A detailed description of the experiment is provided in the supplementary note S1 and ref.^21^.

Here, we experimentally demonstrate for a pulse sequence almost perfect time reversal caused by a temporal modulation of a fibre loop system resulting in population inversion between two briefly touching bands (see Fig. 1). Our proposed protocol relies on the exchange of eigenstates at a band inversion point. Contrary to common observations that closing a band gap by temporal modulation induces an exchange of the eigenstates^16^, we demonstrate that this is not mandatory. Despite of an otherwise adiabatic evolution, a single time step around the band gap closure decides on the kind of non-adiabatic exchange of the eigenvectors and on the quality of time reversal.

## Experimental setup

All measurements are carried out on an experimental platform^21^ (see Fig. 1b and supplementary note 1) consisting of two mutually coupled, but slightly dissimilar fibre loops. In similar experimental realizations of time multiplexed synthetic lattices Anderson localization was studied^22,23^ as well as Boson sampling^24^. The system is widely tuneable in terms of amplitude and phase modulation and capable of depicting linear and nonlinear light evolution^25^. Since the setup is solely based on telecommunication equipment, we want to demonstrate in the following time reversal in an optical fibre system. While the average length of the two loops is about 1 km, the length difference is only approximately 7 m. At the beginning of each measurement, a single pulse is inserted into the longer loop, which can propagate nearly lossless due the use of erbium-doped fibre amplifiers in both loops. Each time a pulse passes the central 50/50 coupler, it splits up into two smaller pulses, which continue propagating in each ring; a process which repeats up to 400 times during the experiment. Due to the length imbalance, pulses arrive at different times at the 50/50 coupler. As long as the loops are not completely filled with the evolving pulse sequence, individual pulses can be uniquely identified by their arrival time

1 $$t=\bar{T}m+n\frac{{\rm{\Delta }}T}{2},$$

which is given by the total number of round trips $m$ in any loop, and the difference $n$ of the number of round trips in the short and long loop. Here, $\bar{T}=({T}_{1}+{T}_{2})/2$ stands for the average transit time, where ${T}_{1}$ and ${T}_{2}$ denote the round trip times of the long and short loop and ${\rm{\Delta }}T\,=\,{T}_{1}-{T}_{2}$ the time delay introduced by the length difference. In our experiment, we are using 25 ns long pulses, which fit into the time window of 35 ns created by the length difference. While using longer pulses would require a larger ${\rm{\Delta }}T$, for much shorter pulses in the picosecond range, dispersion could become a limiting factor.

Based on labelling the pulses by the roundtrip $m$ and the difference $n$ in long loop and short loop roundtrips, the pulse evolution through the fibre loop setup can be mapped on the mesh lattice shown in Fig. 1c: Each row $m$ of the lattice stands for a roundtrip and $n$ is defining the column. In this picture, a roundtrip through the short (long) loop increases $m$ by one and decreases (increases) $n$ by one, leading to a step from North East (West) to South West (East) (see supplementary note 1 and ref.^21^ for a detailed description of the experiment). Mathematically, the evolution is given by a set of two evolution equations^21^

2 $${u}_{n}^{m+1}=\frac{1}{\sqrt{2}}({u}_{n+1}^{m}+i{v}_{n+1}^{m}){e}^{i{\rm{\Phi }}}$$

3 $${v}_{n}^{m+1}=\frac{1}{\sqrt{2}}({v}_{n-1}^{m}+i{u}_{n-1}^{m}),$$

where a phase modulator in the short loop allows for introducing an arbitrary phase shift ${\rm{\Phi }}$.

**Figure 2:**
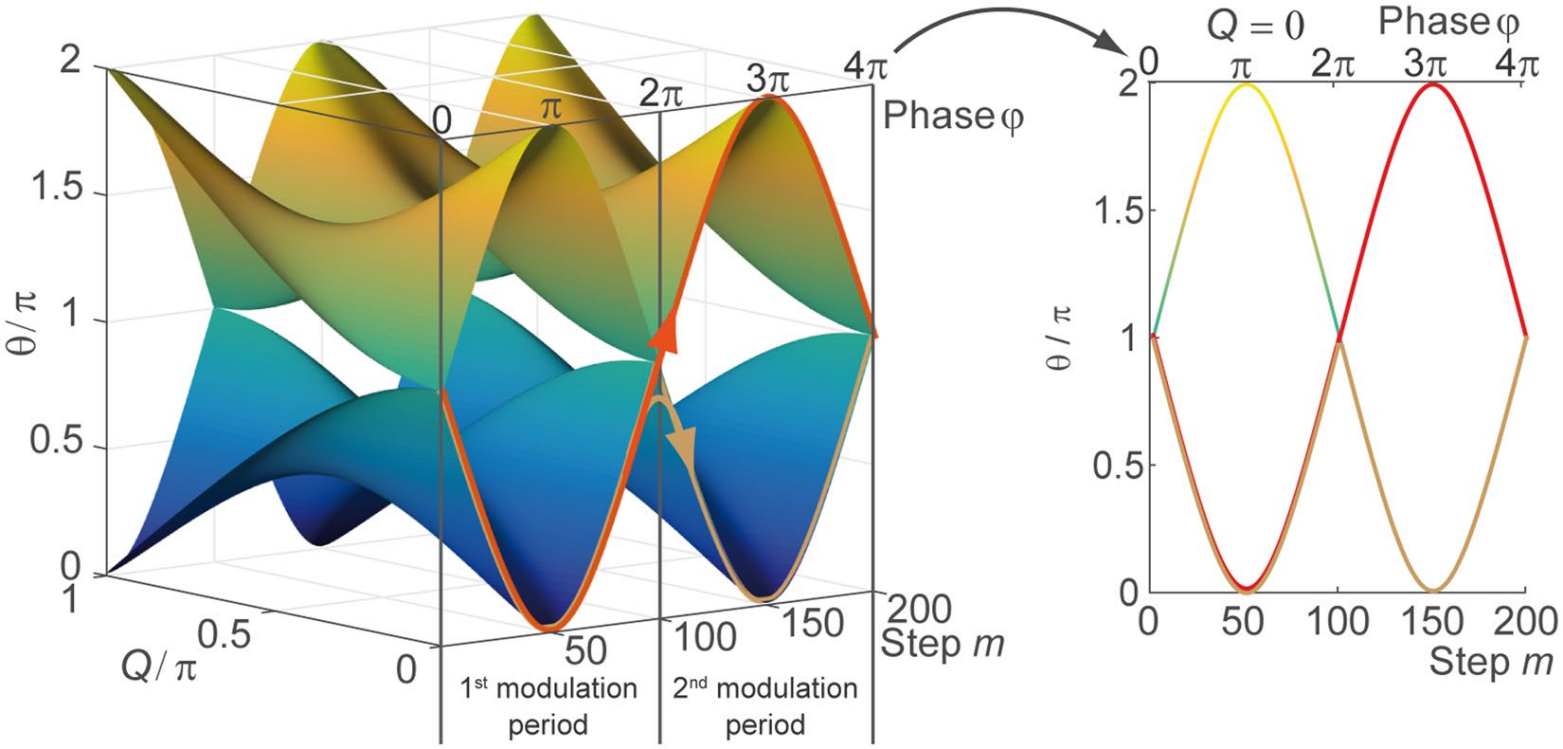
Band structure spanned by the Bloch momentum $Q$ and the temporal driving $m$. For a better visibility, only the parts for $Q\ge 0$ are shown of the otherwise symmetric band structure. For $\phi =k\pi$, the band gaps close at $Q=0$ and $Q=\pi$, which releases the defect to the continuum. Each time the band gap closes, a transition from one band to the other is possible as indicated by the red and brown arrows. The taken path depends on the geometrical relation between the eigenvectors. Changing the band (red curve) inverts the population, and thus starts a time reversal. The right panel depicts the dispersion relation depending on the parameter $\phi$ of the temporal driving for $Q=0$.

For inducing the time reversal of the system, we introduce a temporal modulation

4 $${\rm{\Phi }}(m,n)=\{\begin{array}{l}-\phi ,\,{\rm{mod}}(m,4) < 2\\ +\phi ,\,\mathrm{else}\end{array}$$

of the mesh lattice, where the sign of the phase alternates after every second step. The same modulation scheme was used before to study the influence of the Berry curvature on the wave packet propagation^21^. The dispersion relation of the driven lattice

5 $$4\,\cos \,\theta =\,\cos \,2Q\,-\,\cos \,2\phi -4\,\cos \,Q\,\cos \,\phi $$

is derived using a Floquet-Bloch ansatz

6 $$(\begin{array}{c}{u}_{n}^{m}\\ {v}_{n}^{m}\end{array})=(\begin{array}{c}U\\ V\end{array}){e}^{iQ\frac{n}{2}}{e}^{-i\theta \frac{m}{4}}$$

covering four time steps. The propagation constant $\theta$ in Eq. 5 depends on the Bloch momentum $Q$ as well as on the parameter $\phi$ of the phase modulation in Eq. 4. This allows for tuning the dispersion relation in a desired way and even for closing the band gap at $\phi =k\pi$ with $k\in {{\mathbb{N}}}_{0}$ (see Fig. 2). At a closed band gap, the eigenstates can be exchanged, which inverts the population of the symmetric band structure and consequently the temporal evolution.

## Inversion of the evolution

Injecting a single pulse into one loop excites the whole band structure of the lattice and results in a Light Walk pattern^26,27^, which can be reversed by inverting the evolution operator in Eqs 2 and 3 as demonstrated in ref.^28^. Instead of undoing the evolution of a single pulse, we want to demonstrate a robust method to restore the initial state of a chain of pulses by inverting the population of the underlying band structure (see Fig. 1a for a schematic diagram). For probing the successful restoration of a complex initial wave form, we implement a phase defect^26^ (see method section for details)

7 $${\rm{\Phi }}(m,n)={\phi }_{{\rm{defect}}}({\delta }_{n,0}+{\delta }_{n,+1})+\{\begin{array}{l}-\phi ,\,\mathrm{mod}(m,4) < 2\\ +\phi ,\,{\rm{else}}\end{array}$$

on the lattice at the two central sites $n=0$ and $n=+1$ for all time steps $m$ (corresponding to the rows of the lattice). Such a phase defect supports bound states similar to a quantum mechanical potential well. In almost all cases and also here, bound states are described by propagation constants, which are separated from the bands (also referred to as continuum) and reside inside the band gap to avoid phase matching and energy loss to continuum modes. With increasing defect strength ${\phi }_{{\rm{defect}}}$, the propagation constant moves towards the centre of the band gap, where even a second defect mode appears (see ref.^26^ for details on the defect). For creating a narrow and consequently bright defect mode, we set the defect strength to ${\phi }_{{\rm{defect}}}=\pi .$

**Figure 3:**
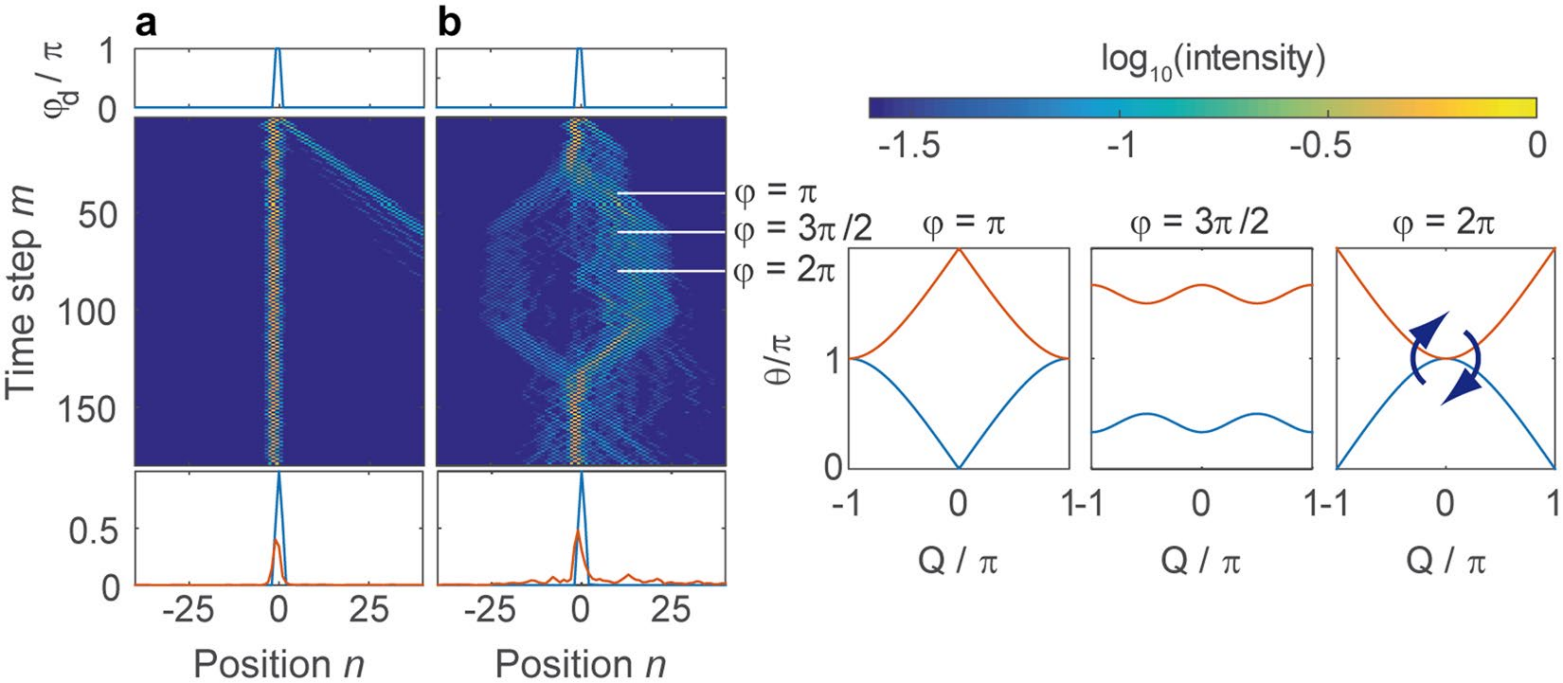
Propagation along a defect with strength ${\phi }_{{\rm{defect}}}=\pi$. (**a**) The defect supports two bound modes showing a mutual beating. (**b**) Combined with the phase modulation in Eq. 7, where $\phi$ is increased by ${\phi }_{0}=2\pi /20\,\,$ after every fourth time step, the band structure is continuously deformed (right panels). When the gap closes, the defect modes couple to the continuum. At $m\approx 80$, when the band gap closes a second time the reversal of the temporal evolution starts, which efficiently restores the initial distribution. In both panels, the intensity ${|{u}_{n}^{m}|}^{2}+{|{v}_{n}^{m}|}^{2}$ in both loops is shown, where the initial pulse injected into the mesh lattice is normalized to one. For a better visibility of the propagation, a logarithmic color scaling is chosen. Below the propagations in (**a**,**b**) the initial intensity distribution ${|{u}_{n}^{0}|}^{2}+{|{v}_{n}^{0}|}^{2}$ (blue curve) and the final distribution ${|{u}_{n}^{179}|}^{2}+{|{v}_{n}^{179}|}^{2}$ (orange curve) at $m=179$ are depicted.

In the experiment, the initial pulse is directly injected at the defect ${n}_{{\rm{initial}}}=0$, which ensures a very efficient excitation of the bound state, i.e. the intensity mainly remains at the lattice sites of the defect. The existence of two bound modes is reflected by a beating of the intensity, which is visible in Fig. 3a for $\phi =0$ (no temporal driving).

For demonstrating the time reversal protocol, we now combine the phase defect with the temporal driving of the system. In the experiment, displayed in Fig. 3b we induce and excite a defect state and perform a global phase modulation according to Eq. 7, thus tuning the band structure around the propagating defect modes. We perform a sweep from $\phi =0$ to $4\pi$ covering two periods of the phase modulation by stepwise increasing $\phi$ after every fourth step by ${\phi }_{0}=2\pi /20$. Induced changes are adiabatic for most of the parameter range as the phase gradient ${\phi }_{0}$ is much smaller than the typical size of the band gap, but adiabaticity necessarily breaks down, when the band gap closes at $\phi =k\pi$. At such a point, when $\phi =\pi$, the propagation constants of the defect modes have to touch the bands and as a consequence, the bound states couple to the bands and the previously guided modes spread in a complex pattern on the lattice (see Fig. 3b, steps $m\approx 40\ldots 120$). Intuitively, one might expect the system to evolve in the same way during the second modulation period starting at $m=80$ (corresponding to $\phi =2\pi$). However, at each gap closure as e.g. for $\phi =2\pi$ at the end of the first modulation period, the system may either stay or switch to the other band which corresponds to an inversion of the population (indicated by the two arrows in Fig. 2). In the latter case, the symmetric band structure mediates the time reversal as visible in the experiment (see Fig. 3b $m > 80$) and also exploited in other time reversal schemes^6^. The originally deliberated field distribution couples back to the defect, where both modes are re-excited and even the beating between them is restored.

## Analysis of the eigenstates

**Figure 4:**
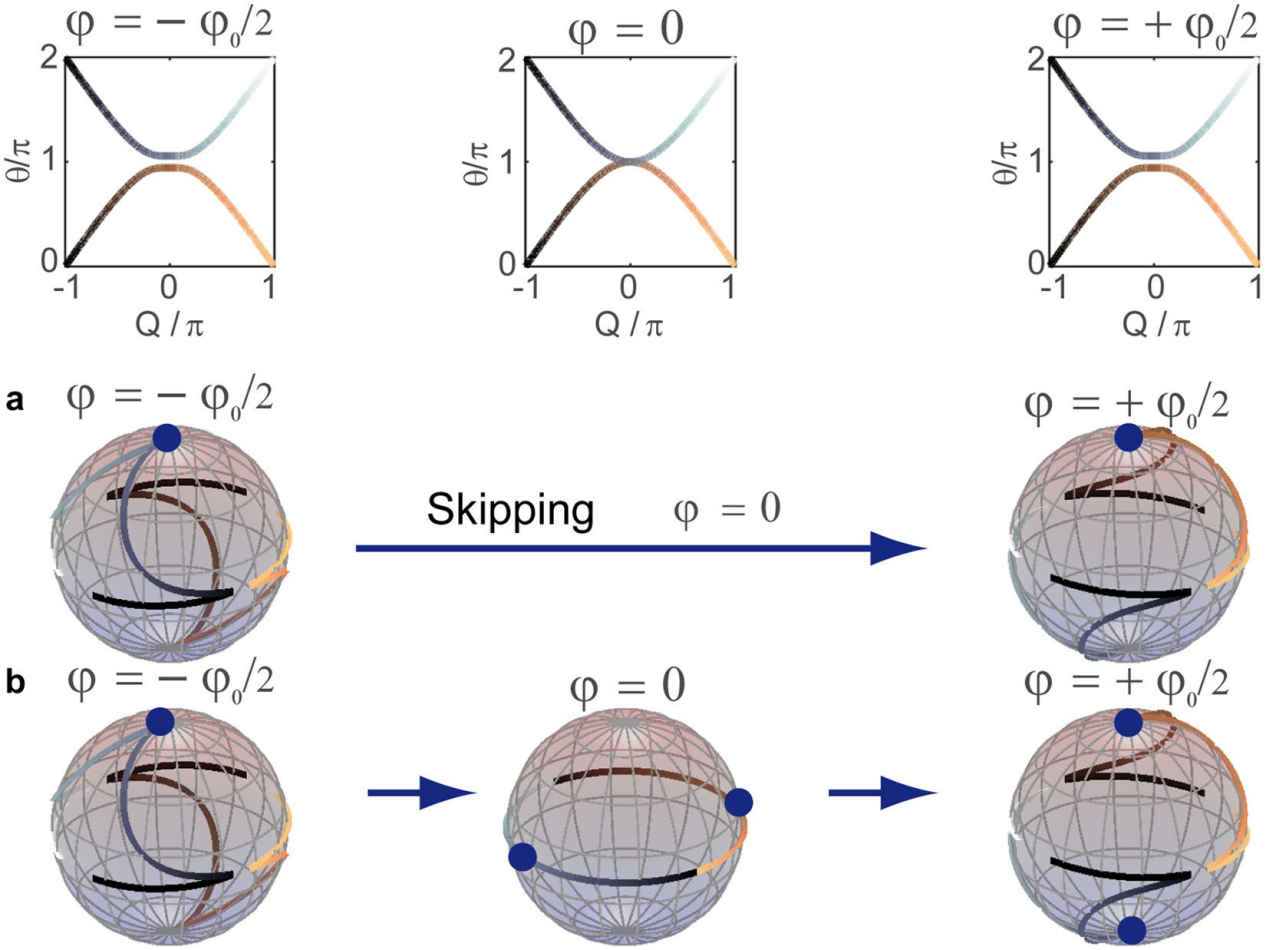
Representation of the complex eigenvectors on the Bloch sphere. Each data point in the dispersion relation and on the Bloch sphere represents a Bloch momentum $Q.$ The shading of the dispersion relation equals the shading of the eigenstates on the Bloch sphere. For $\phi \ne 0$, the eigenstates at $Q=0$ are either located on the North or South pole indicated by the blue circles. When sweeping from $\phi =0$ to $\phi =4\pi$, the singular values $\phi =0,\pi ,2\pi ,\ldots$ can be either avoided or explicitly included. (**a**) If $\phi =0$ is skipped, an excitation of the North pole at $\phi =\,-{\phi }_{0}/2$, will remain on the North pole and change the band, when $\phi$ is set to ${\phi }_{0}/2$. (**b**) If the value $\phi =0$ is in-between included, the excitation is projected at this intermediate step onto the equator, from which it is again equally distributed on the North and South pole. The inclusion of $\phi =0$ is also discussed in Fig. 5. Here, ${\phi }_{0}$ equals $2\pi /25\,$.

But population inversion at gap closure and subsequent time reversal are not mandatory as we will discuss in the following. Since the dispersion relation (see Fig. 2) does not provide the full information, we have to investigate the eigenstates displayed in Fig. 4. For fixed phase parameter $\phi$ and Bloch momentum $Q\,\,$the eigenstates are given by the two component vector $|\psi (Q) > ={(U,V)}^{t}$, which stands for the amplitude and phase relation between both loops. In general this vector is complex, and thus for a better illustration it is depicted on the Bloch sphere by decomposing it into the expectation values (see supplementary note 2)

8 $$\langle {\sigma }_{{\rm{x}},{\rm{y}},{\rm{z}}}(Q)\rangle =\langle \psi (Q)|{\sigma }_{{\rm{x}},{\rm{y}},{\rm{z}}}|\psi (Q)\rangle $$

of the Pauli spin matrices ${\sigma }_{{\rm{x}},{\rm{y}},{\rm{z}}}$. In case of a vanishing phase modulation $\phi =0$, all eigenstates are found on the equator of the Bloch sphere, but even for small phase modulation $\phi =\pm {\phi }_{0}/2$ the eigenstates are tending to the North and South pole for $Q\approx 0$. When flipping the sign of phase modulation both bands exchange the poles, which they originally occupied. To remain in its own band, an eigenstate on the North pole would have to jump to the South pole. Since North and South poles of the Bloch sphere represent orthogonal states, such a sudden jump is forbidden. Hence, if modulation $\phi =0$ is skipped (see Fig. 4a) the eigenstate have to remain on the original pole, and thus perform a transition from one band to the other (see supplementary note 3). As the phase modulation is periodic within $[0;2\pi ]$, this situation not only occurs around $\phi \to \pm 0$, but also around $\phi \to \pm 2\pi$, i.e. at the end of the first modulation period and the beginning of the second. In this sense, the exchange of the eigenstates conclusively explains the reversal of the evolution during the second modulation period.

The band gap not only closes for $\phi =0,2\pi$ but also for $\phi =\pi .$ But in contrast to the previous case eigenstates for $Q\approx 0$, which are dominantly populated by the defect mode are not located on the poles, but instead on the equator. When the phase modulation is swept from $\phi =\pi -{\phi }_{0}/2$ to $\phi =\pi +{\phi }_{0}/2$, the position of those eigenstates remains the same and therefore, no change of the band is induced at this point (see supplementary note 3). It is interesting to note, that always the same temporal driving is applied, but the locations of the eigenstates determine the outcome of the gap closing. As our protocol depends on the exchange of eigenstates, it is mostly independent of the speed of driving as discussed in supplementary note 4. Still this kind of band closure at $\phi =\pi$ has a tremendous effect on the propagating defect mode (see Fig. 3b around $m=40$) as it couples to both bands, but no exchange of bands and no time reversal takes places until the second closure of the gap at $\phi =2\pi$ occurs. At this point the population is inverted, which reverses the evolution of the first period.

## Spectral dependency of the protocol

**Figure 5:**
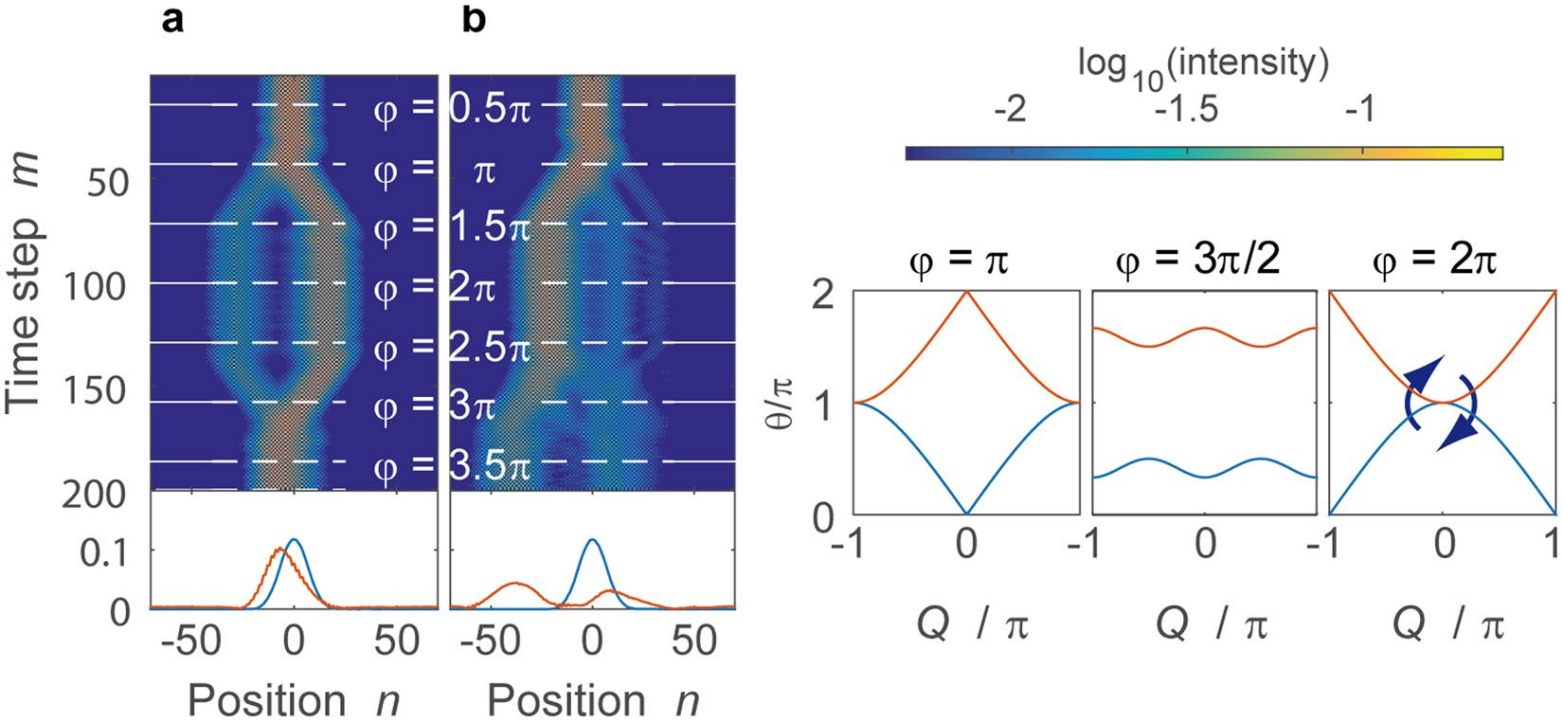
Propagation of a broad wave packet under the influence of the phase modulation given in Eq. 4. The phase $\phi$ is stepwise increased every fourth time step by ${\phi }_{0}=2\pi /25$. (**a**) If the case $\phi =0$ is explicitly excluded the resulting propagation forms a hexagon and the evolution is perfectly reversed, after inverting the population at time step $100$. (**b**) When including $\phi =0$, only one half of the intensity undergoes the time reversal, while the rest is staying the same band repeating the same motion as during the first modulation period. As in Fig. 3, the intensity ${|{u}_{n}^{m}|}^{2}+{|{v}_{n}^{m}|}^{2}$ are shown besides the initial ($m=0$, blue) and final ($m=199\,$, orange) intensity distributions for comparison. The right panels depict the dispersion relation for various values during the motion of the wave packet.

Although the release and subsequent recapture of a defect mode is an impressive demonstration of time reversal, no specific Bloch momentum can be attributed to the defect mode as it covers a broad spectral range. More detailed information on the band structure is gained by using a train of pulses with a broad Gaussian envelope (see supplementary notes 4 and 5) at $Q\approx 0$ with a narrow momentum spread $\delta Q\approx 0.05\pi$ as displayed in Fig. 5. We perform the same time dependent spatially homogenous phase modulation as before according to Eq. 7, but without a localized phase defect (${\phi }_{{\rm{defect}}}=0$). During the propagation, the Gaussian wave packet splits into two counter propagating envelopes as the band structure develops a Dirac cone for $\phi =\pi$. But as long as $\phi =2\pi$ is skipped, time reversal occurs and the initial distribution is restored (see Fig. 5a). Again, this phenomenon is explained by the exchange of $Q\approx 0$ eigenstates between bands at the gap closure at $\phi =2\pi$.

When using tilted beams thus leaving the central part of the Brillouin zone, respective eigenstates are not anymore located on the North and South pole of the Bloch sphere. Consequently, the eigenstates before and after the band closing are not orthogonal anymore resulting in a reduced efficiency of time reversal. As further investigations have shown (see supplementary note 4) our protocol works very well for wave packets covering about the central tenth of the Brillouin zone. Spatially narrow excitations as e.g. the defect mode displayed in Fig. 3 cover a broad range of Bloch momenta and thus do not perform such perfect time reversal as the broad beams investigated in Fig. 5 do. Besides this spectral limitation of the time reversal protocol, a non-ideal phase modulation can reduces the efficiency of our protocol as discussed in supplementary note S4.

An idiosyncrasy of our temporally discretized system is the ability to avoid singular values of the phase modulation, in particular those at $\phi =0,\pi ,2\pi$. Although the exchange of the eigenstates is instantaneous when changing the phase parameter from $\phi =-{\phi }_{0}/2$ to $\phi =+{\phi }_{0}/2$, this step can be artificially split into two by including the value $\phi =0$ (and $\phi =\pi ,2\pi$ accordingly) as discussed in supplementary note 3. For $\phi =0$ the band structure is not only exactly closed but all eigenstates are located on the equator (see Fig. 4b). Consequently, in the first step from $\phi =-{\phi }_{0}/2\to 0$ eigenstates on the upper and lower hemisphere are projected on the equator and in the second step from $\phi =0\to {\phi }_{0}/2$ they are equally redistributed on the upper and lower hemisphere again, thus setting an upper bound of 50% to the efficiency of the time reversal. In the experiment, this is reflected by an imperfect restoration of the wave packet displayed in Fig. 5b, where only half of the intensity is changing the band and restoring the initial wave packet. The other half remains in the original band and just repeats the evolution of the first modulation period.

## Conclusion

In conclusion, a novel scheme of time reversal has been proposed and realized. It is based on an exchange of eigenstates in a two band system causing a band inversion, a concept, which is well known in topology^29^. We further developed this idea using a temporal driving, where the eigenstates are instantaneously projected onto each other at a closed bandgap^16^, the protocol is robust against perturbations as long as only the centre of the Brillouin zone is populated. As the experiment is built up with telecommunication equipment, further applications for pulse switching and steering through optical fibre networks are straight forward. In future, active phase stabilization will be implemented, which allows for injecting an externally created pulse distribution. By placing a pair of coupled fibre loops as discussed in this project on the sender and receiver side of an optical communication link, it is in this way possible to encode and decode optical signals providing an interesting ansatz for time reversal in telecommunication^30^.

## Methods

### Creation of the phase defect

Based on the exact measurement of the fibre loop lengths, it is possible to pre-calculate the arrival times of the pulses at roundtrip $m$ and position $n$ according to Eq. 1. For creating the temporal driving in Eq. 4 and the phase defect in Eq. 7, an electrical signal is produced by an arbitrary waveform generator. The waveform of the signal generator takes the voltage corresponding to a phase shift of $\pi$ during the time frames when the pulses of positions $n=0$ and $n=+1$ arrive at each roundtrip. The internal trigger delay of the waveform generator allows for a synchronisation of the electrical and optical signals.

### Data availability

The data that support the plots within this paper and other findings of this study are available from the corresponding author on request.

## Electronic supplementary material

Supplementary material
