# Supplementary material for: Observation of Time Reversed Light Propagation by an Exchange of Eigenstates
Source: Sci Rep. 2018 Feb 1;8:2125. doi: 10.1038/s41598-018-20577-w (PMC5794902; doi:10.1038/s41598-018-20577-w)
Supplement: Supplementary file 1 — Supplementary material [file 41598_2018_20577_MOESM1_ESM.pdf]

Supplementary Information

Observation of Time Reversed Light Propagation by an Exchange of Eigenstates

Martin Wimmer[1,2] and *Ulf Peschel[2]

[1]Erlangen Graduate School in Advanced Optical Technologies (SAOT), 91058 Erlangen, Germany

[2] Institute of Solid State Theory and Optics, Abbe Center of Photonics, Friedrich-Schiller-Universität Jena, Max-Wien-Platz 1, 07743 Jena, Germany

*Correspondence and requests for materials should be addressed to U.P. (email: ulf.peschel@uni-jena.de)

Supplementary Note 1 - Experimental setup

A detailed description of the experimental setup (see Fig. S1) is provided in [S1,S2]. Each experiment starts with the generation of an initial pulse by cutting the cw signal of a DFB laser diode ($\lambda = 1550$ nm) into a chain of 25 ns pulses. The pulse sequence is amplified twice by erbium-doped amplifiers (EDFA) before it is again shaped by a Mach-Zehnder modulator (MZM) for increasing the background suppression between two adjacent pulses. Finally, the pulses are cleaned by a bandpass filter (BPF) and pass an acousto-optical modulator (AOM). The latter one is set to 100 % transmission during the warm-up of the experiment, i.e. a continuous sequence of pulses enters the experiment, while the AOMs in the loops are preventing any of them from circulating. In this way, the amplifiers in the loop adapt to the input power level, which avoids the occurrence of transients and thus fluctuations of the net gain.

Inside the loops, pilot signals at $\lambda = 1537$ nm are added before the amplifiers, which are filtered out by a tuneable bandpass filter after the EDFAs. The pilot signal allows for a control of the net gain and additionally prevents transients. After the EDFAs, the pulses propagate through 1 km of SSMF fibre before 10 % of the intensity is out-coupled for monitoring the pulse chains. The remaining 90 % travels through isolators preventing back reflections and the build-up of stimulated Brillouin scattering reaching finally the loop AOMs. While these modulators are set to 0 % transmission during the warm-up (full blocking of pulses), they switch to 100 % transmission during the experiment, when the first pulse has entered the loop. At the same time the entrance modulator blocks any further warm-up pulses from entering the loops. Hence, the actual experiment is always performed with a single input pulse only.

In the short loop, a fibre optical phase modulator allows for a controlled phase shift Φ as explained in the main part. At the input of the phase modulator, an integrated polarizer filters out one polarization state. To avoid further losses, polarization controllers are used in the short and long loop for aligning the polarization to achieve maximum transmission through the phase modulator. Additionally, a polarizing beam splitter is inserted into the longer loop, which allows for a better control of the polarization state.

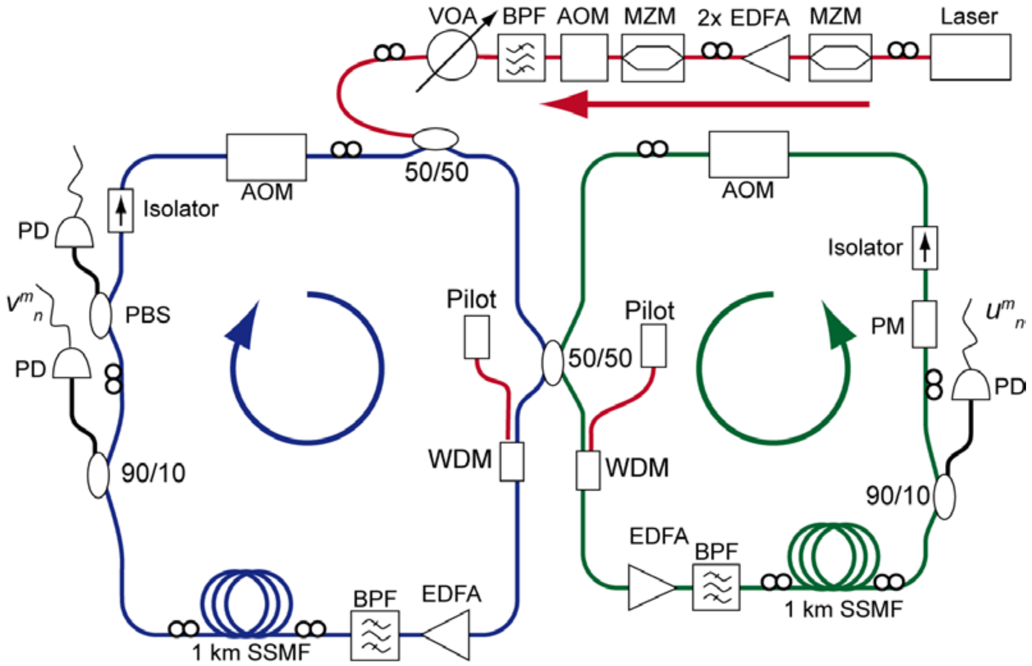

Figure S1. Sketch of the experimental setup adapted from [S1]. Starting from a DFB laser, a chain of 25 ns long pulses is cut out of a cw-signal with a Mach-Zehnder modulator (MZM). The pulses are amplified in the next step by two erbium-doped fibre amplifiers (EDFA) and cut again by a MZM for a strong suppression of background noise. An acousto-optical modulator (AOM) is used as an entrance switch followed by a tuneable bandpass filter (BPF) for cleaning the pulses. A variable optical attenuator allows for a manual control of the peak power. In each loop, the pulses are amplified by an EDFA before they propagate through a 1 km long SSMF fibre. Two 90/10 monitor couplers are used for pulse detection. A polarizing beam splitter (PBS) in the left loop and the phase modulator (PM) in the right loop ensure that only one polarization travels through the loops. Furthermore, the PM allows for an arbitrary phase shift Φ. At the end of each loop, AOMs allow for an amplitude modulation as described in [S2], which is needed for shaping Gaussian wave packets and for blocking the warm-up pulses.

Supplementary Note 2 - Eigenstates and Bloch sphere representation

According to the Floquet-Bloch ansatz in Eq. 6 the eigenstates are given by $|\psi(Q)>=(U,V)^{\mathrm{t}}$, which can be also expressed by

$$|\psi(Q)>=\frac{1}{\sqrt{1+|\alpha_{\pm}(Q)|^2}}\begin{pmatrix}1\\ \alpha_{\pm}(Q)\end{pmatrix}, \tag{S1}$$

where

$$\alpha_{\pm}(Q)=\frac{-4\mathrm{e}^{\mp i\theta}+\mathrm{e}^{2iQ}-\mathrm{e}^{2i\varphi}-2\mathrm{e}^{iQ+i\varphi}-\mathrm{e}^{-iQ+i\varphi}-\mathrm{e}^{iQ-i\varphi}}{i\left(e^{2iQ}+e^{2i\varphi}+e^{-iQ+i\varphi}-e^{iQ-i\varphi}-2\right)} \tag{S2}$$

is a complex number depending on Q,φ and on whether the upper or lower band is chosen. For better depicting the arrangement of the eigenstates in the complex plane, their location on the Bloch sphere is shown in Fig. S2. In this plot, the coordinates of each point are given by the three expectation values $\vec{n}=\left(\langle\sigma_{\mathrm{x}}\rangle,\langle\sigma_{\mathrm{y}}\rangle,\langle\sigma_{\mathrm{z}}\rangle\right)^{\mathrm{t}}$ of the Pauli matrices

$$\sigma_{\mathrm{x}}=\begin{pmatrix}0 & 1\\ 1 & 0\end{pmatrix}, \tag{S3}$$

$$\sigma_{\mathrm{y}}=\begin{pmatrix}0 & -i\\ i & 0\end{pmatrix}, \tag{S4}$$

$$\sigma_{z}=\begin{pmatrix}1 & 0\\ 0 & -1\end{pmatrix}. \tag{S5}$$

For an arbitrary eigenstate given by Eq. S1 the expectation values take the form

$$\langle\sigma_{\mathrm{x}}\rangle=\frac{1}{1+|\alpha_{\pm}|^2}\begin{pmatrix}1 & \alpha_{\pm}^*\end{pmatrix}\sigma_{\mathrm{x}}\begin{pmatrix}1\\ \alpha_{\pm}\end{pmatrix}=\frac{2\mathrm{Re}(\alpha_{\pm})}{1+|\alpha_{\pm}|^2} \tag{S6}$$

$$\langle\sigma_{\mathrm{y}}\rangle=\frac{1}{1+|\alpha_{\pm}|^2}\begin{pmatrix}1 & \alpha_{\pm}^*\end{pmatrix}\sigma_{\mathrm{y}}\begin{pmatrix}1\\ \alpha_{\pm}\end{pmatrix}=\frac{2\mathrm{Im}(\alpha_{\pm})}{1+|\alpha_{\pm}|^2} \tag{S7}$$

$$\langle\sigma_{\mathrm{z}}\rangle=\frac{1}{1+|\alpha_{\pm}|^2}\begin{pmatrix}1 & \alpha_{\pm}^*\end{pmatrix}\sigma_{\mathrm{z}}\begin{pmatrix}1\\ \alpha_{\pm}\end{pmatrix}=\frac{1-|\alpha_{\pm}|^2}{1+|\alpha_{\pm}|^2} \tag{S8}$$

with $\langle\sigma_{\mathrm{x}}\rangle^2+\langle\sigma_{\mathrm{y}}\rangle^2+\langle\sigma_{\mathrm{z}}\rangle^2=1$. When directly evaluating Eqs. S6, S7 and S8 for the passive case $\varphi=0$, an intrinsic rotation of the eigenstates is visible (see Fig. S2a), which is compensated by a rotation of $\pi/4$ about the σ_{x} axis (see Fig. S2b)

$$\vec{n}'=\begin{pmatrix}1 & 0 & 0\\ 0 & \cos\frac{\pi}{4} & \sin-\frac{\pi}{4}\\ 0 & \sin\frac{\pi}{4} & \cos\frac{\pi}{4}\end{pmatrix}\begin{pmatrix}\langle\sigma_{\mathrm{x}}\rangle\\ \langle\sigma_{\mathrm{y}}\rangle\\ \langle\sigma_{\mathrm{z}}\rangle\end{pmatrix} \tag{S9}$$

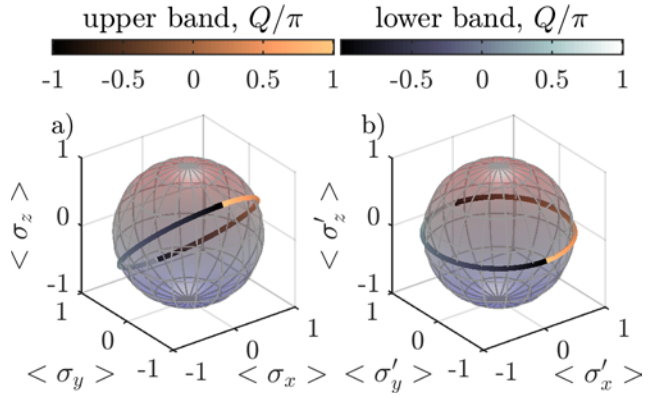

Figure S2. For a better visualization, the eigenstates are decomposed into the expectation values of the Pauli matrices in Eqs. S6, S7, S9 and plotted on the Bloch sphere. For $\varphi = 0$, all states are lying on a great circle on the Bloch sphere. The intrinsic misalignment is fixed by a $\pi/4$ rotation about the x-axis described by Eq. S9 so that finally all eigenstates occupy the equator. The shading of the lines shows the Bloch momentum Q.

Supplementary Note 3 – Transition form the upper to the lower band

In presence of a non-vanishing phase modulation the eigenstates are not anymore in-plane as shown in Fig. S2b, but instead for $\varphi \to 0$ they are distributed on the upper and lower half of the Bloch sphere (see Fig. S3). Furthermore, for $Q = 0$ the states are found close to the North and South pole depending on the band and the sign of φ. By switching the latter one, both bands swap their positions on the Bloch sphere, and thus the eigenstates at $Q = 0$ either have to change the band or the pole. Since the states on the North and South poles are orthogonal to each other, switching between the poles is highly suppressed, and thus an excitation at $Q = 0$ must change the band in order to remain on the original pole.

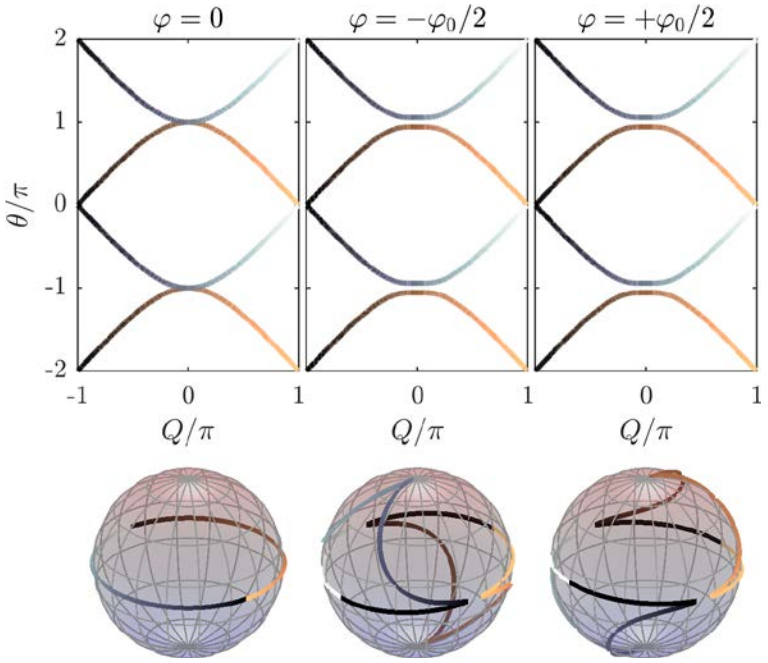

Figure S3. Dispersion relation and eigenstates for different values of φ. Due to the Floquet-Bloch ansatz in Eq. 6, the band structure is periodic in θ and Q. For $\varphi = 0$ all eigenstates are found on the equator independent of their Bloch momentum Q (see Fig. S2b). In presence of a small phase shift $\varphi_0 = 2\pi/25$, the eigenstates for $Q = 0$ are tending to the North (lower band) and South Pole (upper band) of the Bloch sphere. When switching the sign of φ a wave packet at $Q = 0$ either has to stay in the same band, which requires a transition from one pole to the other, or alternatively change from one band to the other in order to remain on the original pole and thus in the original eigenstate. In case of $\varphi = 0$, the band gaps are closed due to the Floquet nature of the system. However, for any non-vanishing $\varphi \neq 0$, a finite gap separates the two bands.

Compared to the situation of φ close to $0, 2\pi, 4\pi, \ldots$, the case of φ around $\pi, 3\pi, 5\pi, \ldots$, which also features a closed band gap, differs significantly (see Fig. S4): Here, the eigenstates at $Q = 0$ are on the equator, while the states at $Q = \pm\pi$ occupy the poles. Since, the eigenstates at $Q = 0$ stay invariant under the transition from $\pi - \varphi_0/2 \to \pi + \varphi_0/2$, a wave packet at $Q = 0$ will stay in its original band. Consequently, for a wave packet at $Q = 0$ the

band transition only takes place at $\varphi = 0{,}2\pi, \dots$, which is in excellent agreement with the experimentally observed time reversed evolution shown in Figs. 3 and 5 during the second modulation period.

In general, the band structure is symmetric with respect to the transformation $Q \to Q + \pi$, when simultaneously shifting φ by π. Therefore, this discussion is also valid for the edge of the Brillouin zone, i.e. a wave packet at $Q = \pm\pi$ will also perform a time reversal, but for $= \pi, 3\pi, \dots$.

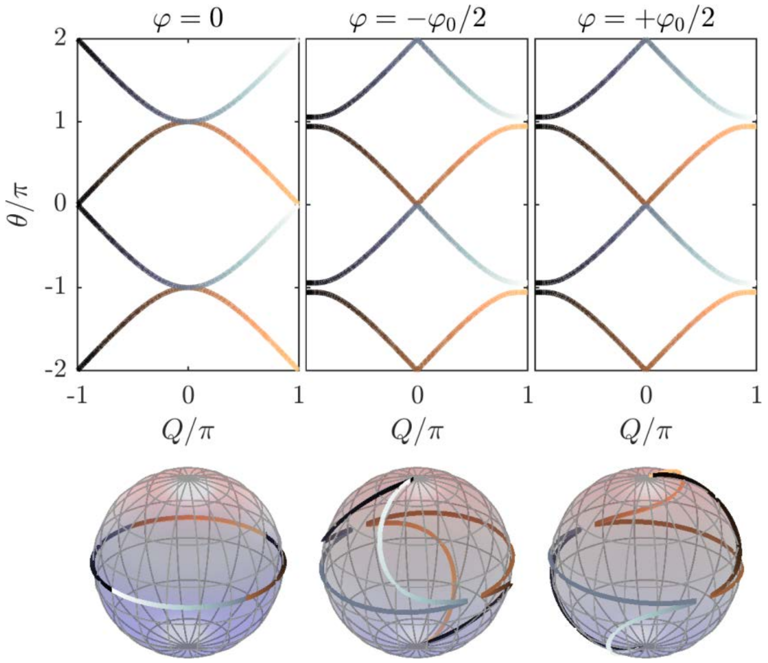

Figure S4. Same situation as in Fig. S3 centred at $\varphi = \pi$. Also here a band gap opens at $Q = \pm\pi$. However, the eigenstates at $Q = 0$ are located on the equator and do not change before and after the gap closing. Consequently, no transition takes place for wave packets centred at $Q = 0$ and $\varphi = \pi$.

Finally, the pathologic case of $\varphi = 0$ plays a central role for the time reversal. If this case is included, the eigenstates are first projected onto the equator for $\varphi = -\varphi_0/2 \to 0$. In the next step, from $\varphi = 0 \to \varphi_0/2$ they are again redistributed on the upper and lower poles of the Bloch sphere. However, as the scalar products between states on the equator and states on either the North or South Pole are equal, the wave packet is also equally distributed between the poles. Therefore, half of the excitation changes the band and reverses the evolution, while the other half remains in the original band and continuous the propagation as before.

In order to exclude the case $\varphi = 0$ and to maximize the time reversal efficiency, it has to be ensured that the phase modulation

$$\Phi = \begin{cases} -\varphi, & \mathrm{mod}(m, 4) < 2 \\ +\varphi, & \mathrm{else} \end{cases} \tag{S10}$$

is free of $\varphi = 0$. For this reason, the parameter φ is stepwise increased after every fourth time step according to

$$\varphi(m) = \varphi_0 \left(\left\lfloor \frac{m}{4} \right\rfloor - \delta \right). \tag{S11}$$

Here, $\lfloor . \rfloor$ denotes the floor function and δ is an offset, which allows for avoiding $\varphi = 0$. When δ is set to $1/4$, it is guaranteed, that during the modulation cycle the state $\varphi = 0$ is skipped. For this reason, δ is set to $1/4$ for all measurements shown in the manuscript except for Figs. 5b, which explicitly depicts the inefficient time reversal by hitting the case to $\varphi = 0$. In the same way, the parameter δ is also used in the simulation to avoid or exactly include $\varphi = 0$ (see Fig. S9b).

Supplementary Note 4 - Transition probability

As discussed in supplementary note 3, it depends on the initial Bloch momentum Q whether a time reversal takes place or not. While the cases $Q \approx 0$ and $Q \approx \pm\pi$ were investigated before, the following discussion focuses on arbitrary Q between 0 and $\pm\pi$. For quantifying the efficiency of the population inversion, the geometric relation before and after the gap closing has to be studied. As the phase modulation keeps the Bloch momentum Q invariant throughout the full modulation period, only the transition from one eigenstate before the gap closing to the two eigenstates with the same Bloch momentum Q after the gap closing has to be considered. Here, we denote the initial state by

$$|\psi_{\text{before}}(Q)>=\frac{1}{\sqrt{1+|\alpha_{\text{before}}(Q)|^2}}\begin{pmatrix}1\\ \alpha_{\text{before}}(Q)\end{pmatrix} \tag{S12}$$

and the two final eigenstates for the upper (+) lower band (−) by

$$\psi_{\text{after},\pm}(Q)>=\frac{1}{\sqrt{1+|\alpha_{\text{after},\pm}(Q)|^2}}\begin{pmatrix}1\\ \alpha_{\text{after},\pm}(Q)\end{pmatrix} \tag{S13}$$

The percentage of light, which performs a transition from one band to the other can be approximated by the Hermitian scalar product

$$P=\left|\left\langle\psi_{\text{before}}(Q)\middle|\psi_{\text{after},\pm}(Q)\right\rangle\right|^2 \tag{S14}$$

between the eigenstates before and after the gap closing. The experiment as well as the theoretical discussion focuses on classical light propagation in absence of any quantum mechanical phenomena. Eq. S14 only reflects the geometrical relation of the eigenstates on the Bloch sphere, where $P=0$ for two orthogonal eigenstates and $P=1$ for two parallel eigenstates.

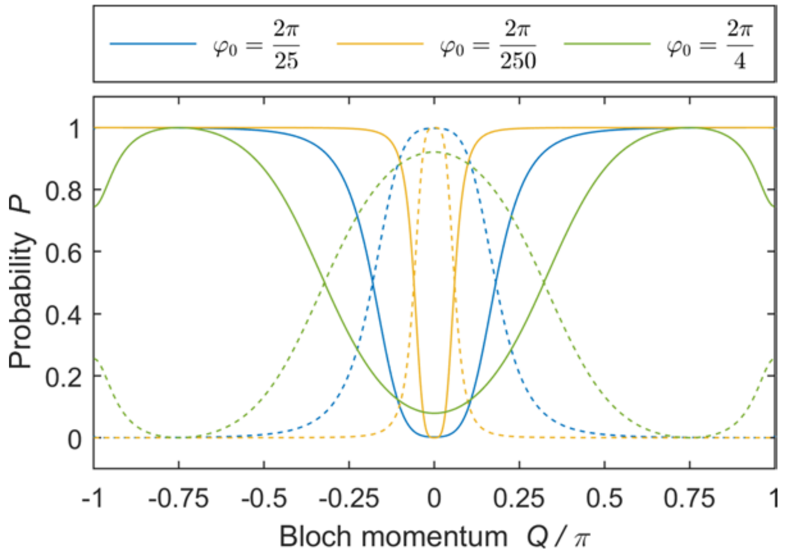

Figure S5. Transition efficiency according to Eq. S14 for a gap closing at φ_0. The efficiency for staying in the same band is shown as a bold line, while the efficiency for changing the band is shown as a dashed line. The different colors depict three different modulation speeds φ_0.

Based on Eq. S14 it is now possible to calculate a classical transition efficiency by evaluating the scalar product numerically for different Bloch momenta Q (see Fig. S5). For a very small gradient $\varphi_0 = 2\pi/250$ (yellow curve), where the evolution is even close to the gap still in the adiabatic regime, the efficiency for a transition from one band to the other at $Q \approx 0$ is very high $P \approx 1$. However, at $Q \approx 0.06\pi$ the efficiency is already dropping to 50 % for the same gradient. With increasing gradient the interval of Bloch momenta widens for which a high efficient time reversal can be expected. For the parameter $\varphi_0 = 2\pi/25$, which was used in the experiment, the efficiency at $Q = 0$ is still tending to $P \approx 1$, while the efficiency drops to 50 % at $Q \approx 0.18\pi$. Finally, for very large modulation steps of $\varphi_0 = 2\pi/4$, the 50 % drop occurs at $Q \approx 0.32\pi$, while the maximum efficiency is about 92 %.

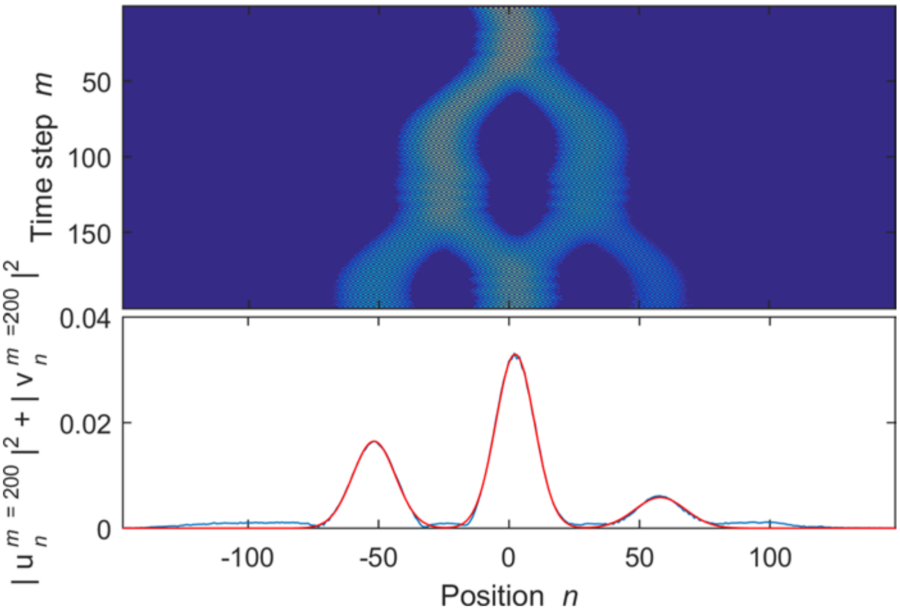

Figure S6. Example for a measured wave packet propagation with narrow momentum spread launched at $Q = -0.2\pi$. Most of the intensity restores the initial distribution in the last row $m = 200$ at $n \approx 0$. However, as the transition efficiency is below 100 % (see Fig. S5), parts of the initial wave packet propagate during the second half from $m = 100$ to $m = 200$ as during the first modulation period ($m = 0$ to $m = 100$) and thus separate further from the centre. These parts are not time reversed in contrast to the central peak at $n = 0$. The lower panel shows the intensity averaged over the last four time steps to which three Gaussian distributions according to Eq. S15 are fitted.

In the experiment, the transition efficiency is measured by launching a broad wave packet with a well-defined Bloch momentum Q_{initial} and narrow momentum spread $\delta Q \approx 0.05\pi$ (see Fig. S6). For $Q = 0$ the propagation forms a hexagon as discussed in the main part, where due to the perfect time reversal the initial wave packet is restored. By evaluating the amount of light, which is found at the initial location, the overall efficiency can be estimated. In the same way, the inefficiency is measured by the wave packet branches, which are further separating from the centre during the second modulation period, since they don't undergo the time reversal. In order to cover all three cases, a superposition of three Gaussian distributions

$$f(n) = A_1 e^{-\frac{(n-n_1)^2}{c_1^2}} + A_2 e^{-\frac{(n-n_2)^2}{c_2^2}} + A_3 e^{-\frac{(n-n_3)^2}{c_3^2}} \tag{S15}$$

is fitted to the intensity averaged over the last four time steps (see Fig. S6 lower panel). While one distribution is initialized at $n_1 = 0$ measuring the time reversed amount, the initial conditions of the two other contributions are set to $n_{2,3} = \pm 50$. The power, which is covered by the central Gaussian distribution, is given by

$$P_{\text{transition}} \approx \int_{-\infty}^{\infty} A_1\, e^{-\frac{(n-n_1)^2}{c_1^2}} \mathrm{d}n = \sqrt{\pi} A_1 c_1 \tag{S16}$$

in the continuous limit. While Eq. S16 determines the efficiency for the band transition, the inefficiency, i.e. the amount which stays in the original band, is given by

$$P_{\text{no transition}} \approx \sqrt{\pi} A_2 c_2 + \sqrt{\pi} A_3 c_3 \tag{S17}$$

A parameter sweep of Q from -0.4π to 0.4π in steps of $\Delta Q = 0.05\pi$ is performed in order to spectrally resolve the time reversal efficiency. The results are shown in Fig. S7 and show a qualitatively good agreement between theory and experiment. Main deviations are a result of the background noise, which is also integrated by Eq. S16.

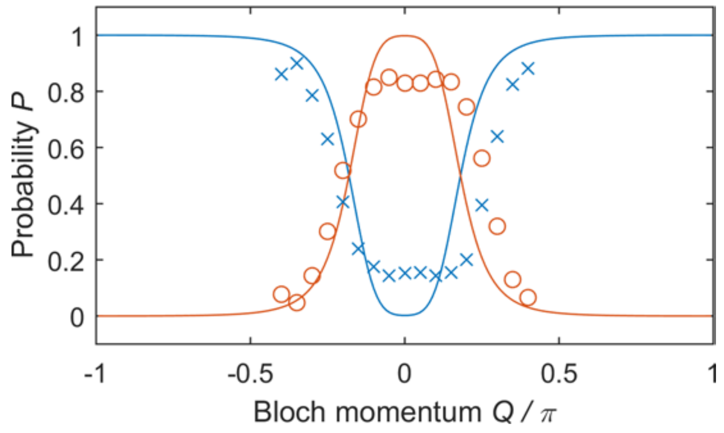

Figure S7. Experimentally obtained efficiencies for wave packets with a momentum spread of $Q \approx 0.05\pi$ centered at the initial Bloch momentum Q. The efficiency is estimated according to Eq. S16 and marked by orange circles, while the inefficiency is shown by blue crosses and estimated by Eq. S17. Solid lines mark theoretical expectations based on Eq. S14.

Supplementary Note 5 - Excitation of broad wave packets and comparison to simulations

At the beginning of every measurement, only a single laser pulse is inserted at $n = 0$. In case that a broad wave packet is needed as e.g. for investigating the evolution of a broad wave packet (see Fig. 5 and supplementary note S4), every second time step either the long or short loop is completely blocked. This results in a diffusive evolution of the wave packet as shown in Fig. S8 until time step 172. The final pulse sequence has a Gaussian shape with flat phase. By modulating the phase in the final step, a specific band is selected and a certain mean momentum can be attributed to the wave form. The resulting Gaussian field distribution is used as an initial state for further measurements. From step 172 on, no loops are further blocked but instead the phase modulator is used for temporally driving the lattice following the scheme described in the main paper. A detailed description of this procedure is provided in [S2].

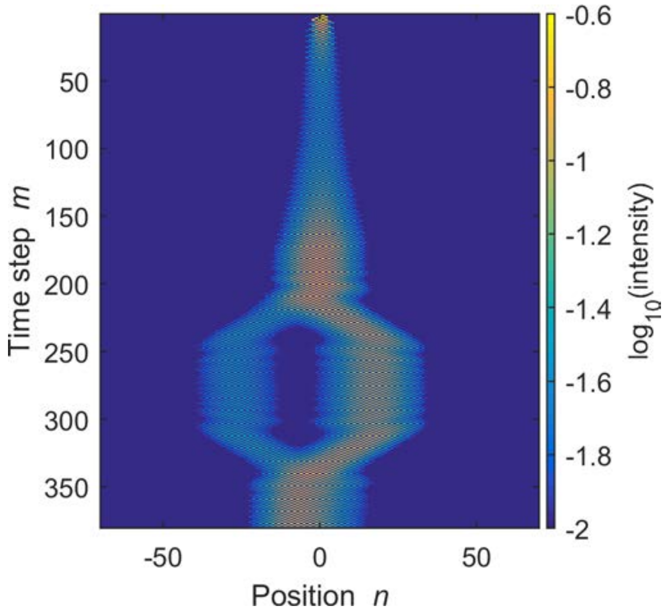

Figure S8. At the beginning of the experiment, a single pulse is inserted, which spreads diffusively when blocking one of the two loops every second time step. From time step $m = 172$ on, none of the loops is blocked anymore. Instead, the propagation of the wave packet is modulated by the temporally phase modulation for closing and reopening the band gap. In the other figures, the preparation of the initial wave packet is not depicted but instead only the relevant propagation from $m = 172$ to $m = 372$ is shown.

Figure S9 depicts a simulated propagation for a wave packet launched at the upper band at $Q = 0$ with a width of eight positions. The parameters are matching those of the experiment, which are shown in Fig. 5a,b of the main part. The agreement between the experiment and simulation is excellent and demonstrates the fundamental influence of the case $\varphi = 0$ for the wave packet evolution.

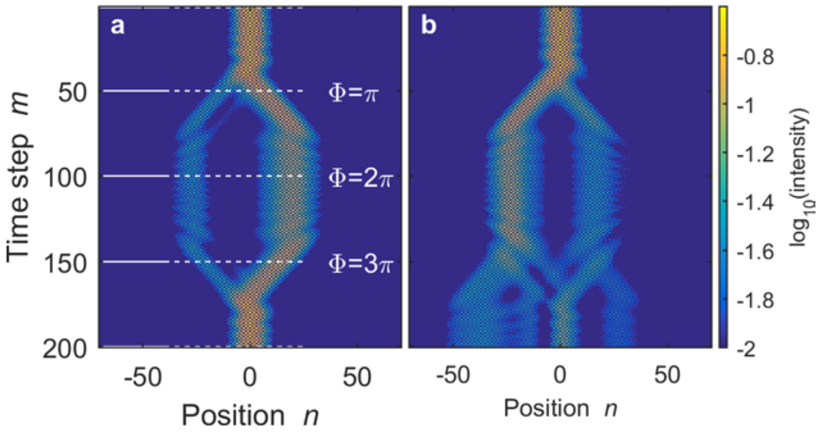

Figure S9. Simulated propagation of the wave packet in presence of the phase modulation. a, When avoiding $\Phi = 0, 2\pi, \ldots$ by including the offset $\delta = 0.25$, the hexagon is perfectly restored. b, However, by explicitly including $\Phi = 2\pi$, the efficiency of the time reversal is strongly hindered $(\delta = 0)$. Only 50% of the intensity tends back to the centre, while the remaining intensity moves to the left and right.

Supplementary References:

S1. Wimmer, M., Price, H. M., Carusotto, I. & Peschel, U. Experimental measurement of the Berry curvature from anomalous transport. *Nat. Phys.* **13,** 545–550 (2017).

S2. Wimmer, M. et al. Optical diametric drive acceleration through action–reaction symmetry breaking. Nat. Phys. **9,** 780–784 (2013).
